# Trends in incidence, prevalence, and mortality of peptic ulcer disease in Asia (1990–2023) and projections to 2040

**DOI:** 10.1097/MD.0000000000050833

**Published:** 2026-09-25

**Authors:** Siying Huang, Kaibin Fang, Weitao Hu, Dunhuang Peng

**Affiliations:** a Department of Gastroenterology, The Second Affiliated Hospital of Fujian Medical University, Quanzhou, Fujian, P.R. China.

**Keywords:** Asia, disease burden, incidence, peptic ulcer disease, prevalence

## Abstract

**Background:**

This study provides the first comprehensive assessment of the burden of peptic ulcer disease (PUD) in Asia, analyzing epidemiological trends from 1990 to 2023 with projections to 2040.

**Methods:**

Using data from the Global Burden of Disease Study 2023, we analyzed PUD metrics across 48 Asian countries and territories. Age-standardized rates were calculated by the direct method. Temporal trends were evaluated by Joinpoint regression, and future burden was projected to 2040 using autoregressive integrated moving average (ARIMA) modeling.

**Results:**

In 2023, Asia recorded 1.87 million incident PUD cases (age-standardized incidence rate: 39.34 per 100,000), 4.32 million prevalent cases (age-standardized prevalence rate: 90.58 per 100,000), and 131,011 deaths (age-standardized death rate: 2.85 per 100,000). PUD caused 3.46 million Disability-adjusted life years, with premature mortality accounting for >90% of the total burden. From 1990 to 2023, Joinpoint regression revealed significant declining trends across all age-standardized metrics (annual percentage change range: −1.32%–− 3.27%; all *P* < .05). ARIMA projections to 2040 suggest continued decreases in all rates, with years lived with disability expected to exceed years of life losts as the dominant component of disease burden.

**Conclusion:**

Despite declining incidence, PUD continues to impose a substantial disease burden across Asia. The persistent toll: driven by an expanding and aging population: underscores the need for sustained prevention efforts, including population-based Helicobacter pylori screening, rational non-steroidal anti-inflammatory drugs prescribing, and health system capacity for emergency endoscopic intervention and long-term chronic disease management.

## 1. Introduction

Peptic ulcer disease (PUD) refers to chronic ulcers of the gastric or duodenal mucosa caused by local digestive action; at its core, it is a circumscribed tissue defect resulting from “self-digestion” of the mucosa that extends at least to the muscularis mucosae.^[1]^Ulcer formation occurs when aggressive factors: such as gastric acid and pepsin: overwhelm the defensive capacity of the gastro-duodenal mucosa.^[2]^ Multiple factors contribute to PUD, the most important being Helicobacter pylori infection and the use of certain drugs.^[3]^ Helicobacter pylori is the commonest cause, accounting for more than half of all ulcers.^[4]^ The bacterium disrupts mucosal defenses and may alter gastric hormone release, leading to abnormal acid output that predisposes to ulceration.^[5]^ Chronic or high-dose use of aspirin, ibuprofen, naproxen, and other non-steroidal anti-inflammatory drugs (NSAIDs) is another major etiology; these agents impair mucosal protection and repair by inhibiting prostaglandin synthesis.^[6]^ Physiologic stress from extensive burns, head trauma, critical illness, or major surgery can precipitate stress-related ulcers.^[7]^ Cigarette smoking and excessive alcohol ingestion exacerbate symptoms and delay healing.^[8]^ Zollinger-Ellison syndrome, characterized by profound gastric acid hypersecretion, also causes ulcer disease.^[9]^

Since the early 21st century, the global incidence of PUD has been declining.^[10]^ Yet in Asia, PUD still imposes a substantial disease burden. In China, the prevalence among adults is close to 10%,^[11]^ and many patients are hospitalized for complications such as perforation or bleeding.^[12]^ In Korea, the overall number of PUD cases has remained unchanged, while the ulcer disease in the elderly is rising.^[13]^ Perforated peptic ulcers in India account for appreciable morbidity and mortality.^[14]^ Globally, both incidence and mortality from PUD have fallen markedly, but in 2019 South Asia still carried the world’s highest prevalence.^[15]^ Understanding the disease burden and epidemiological trends of PUD across Asia and within individual countries is essential for designing targeted prevention and control strategies.^[16]^

The Global Burden of Disease (GBD) Study: led by the Institute for Health Metrics and Evaluation at the University of Washington: tracks the health impact of 375 diseases (including PUD) and 88 risk factors across 204 countries and territories worldwide.^[17]^ While previous GBD-based analyses have examined the global burden of PUD or focused on specific regions such as China, South Korea, or South Asia, no study to date has provided a comprehensive, continent-wide assessment of PUD epidemiology across all Asian countries and territories. Using the newly released GBD 2023 database, we conducted a secondary analysis to quantify PUD burden indicators for 48 Asian countries and territories, providing the first comprehensive assessment of the disease burden and epidemiological trends of PUD across the entire Asian continent.

## 2. Methods

### 2.1. Data sources

GBD 2023 pools data from household surveys, civil registration, censuses, and routine health-service records into 1 modeling platform. The unified methods guarantee that estimates can be compared across countries, yielding incidence, prevalence, and other core metrics. Diseases are mapped to ICD codes, ensuring consistent and detailed tracking of population-health trends.

As the GBD database does not list “Asia” as a ready-made region, we pulled the PUD estimates for every Asian country and territory individually, following the GBD regional sequence. The extracted countries include Afghanistan, Armenia, Azerbaijan, Bahrain, Bangladesh, Bhutan, Brunei Darussalam, Cambodia, China, Cyprus, Democratic People’s Republic of Korea, Georgia, India, Indonesia, Iran (Islamic Republic of), Iraq, Israel, Japan, Jordan, Kazakhstan, Kuwait, Kyrgyzstan, Lao People’s Democratic Republic, Lebanon, Malaysia, Maldives, Mongolia, Myanmar, Nepal, Oman, Pakistan, Palestine, Philippines, Qatar, Republic of Korea, Saudi Arabia, Singapore, Sri Lanka, Syrian Arab Republic, Taiwan (Province of China), Tajikistan, Thailand, Timor-Leste, Turkey, Turkmenistan, United Arab Emirates, Uzbekistan, Viet Nam, Yemen.

Based on the “Cause of death or injury” category in the GBD 2023 database, we extracted disease burden of PUD for 20 age groups: “<5,” “5 to 9,” “10 to 14,” “15 to 19”, 20 to 24,” “25 to 29,” “30 to 34,” “35 to 39”, 40 to 44,” “45 to 49,” “50 to 54,” “55 to 59”, 60 to 64,” “65 to 69,” “70 to 74,” “75 to 79”, 80 to 84,” “85 to 89,” “90 to 94,” “95 plus.”

### 2.2. Statistical analysis

Data on the incidence, prevalence, death, Years of Life Lost (YLL), disability-adjusted life years (DALYs) and years lived with disability (YLDs) for PUD across 48 Asian countries and territories were obtained from the GBD 2023 study. Subsequently, we calculated the age-standardized rates for incidence (age-standardized incidence rate [ASIR]), prevalence (age-standardized prevalence rate [ASPR]), Deaths (age-standardized death rate [ASDR]), DALYs, YLLs, and YLDs, reporting each estimate along with its corresponding 95% uncertainty interval (UI).

### 2.3. Age-standardized rates

To level the playing field across countries and years, every metric was converted into an age-standardized rate per 100,000 using the direct method (epitools::ageadjust.direct). This strips away the influence of shifting age pyramids and yields directly comparable ASIR, ASPR, ASDR, and corresponding DALY, YLL and YLD rates. Raw counts are nevertheless reported alongside these adjusted figures so that planners can see both the relative burden and the absolute numbers that drive service needs. All age-standardized calculations relied on population data obtained from the GBD 2023 demographic resource.

We first added up the age-group populations of the 48 Asian countries/territories, then reweighted the totals to the global standard population structure before standardizing the rates.

### 2.4. Average annual percent change

Joinpoint regression was applied to each age-standardized rate series (ASIR, ASPR, ASDR, age-standardized DALY, YLL, and YLD rates) to estimate the Average Annual Percentage Change (AAPC) with 95% confidence intervals (CIs), with the maximum number of joinpoints constrained to ensure model parsimony. All analyses were performed using the Joinpoint Regression Program (version 5.1.0), a specialized statistical software package designed for piecewise linear trend analysis. This program identifies the optimal joinpoint model through an iterative algorithm that evaluates user-specified constraints on the minimum and maximum number of joinpoints. To adequately capture potential trend inflections during the 1990 to 2023 study period, the maximum number of joinpoints was set to 5. Consistent with the National Cancer Institute’s recommendations, the optimal model was selected via Monte Carlo permutation tests (4499 permutations, α = 0.05). Heteroscedasticity was addressed through weighted least squares regression, with weights specified as the inverse of the estimated variance of each age-standardized rate, thereby appropriately downweighting less precise annual estimates.

### 2.5. Autoregressive integrated moving average

To project the disease burden of PUD in Asia through 2040, we employed an ARIMA model: a well-established approach in health research that integrates autoregression (AR), differencing (I), and moving average (MA) components to capture temporal trends, seasonal patterns, and stochastic fluctuations in time-series data. All time-series analyses and forecasting were performed in R (version 4.x) using the forecast package (version 8.22). Model parameters (p, d, q) were automatically selected via the auto.arima() function, which identifies the optimal model specification by minimizing the corrected Akaike Information Criterion (AICc) to prevent overfitting. To ensure a comprehensive and unbiased parameter search, both stepwise optimization and approximation algorithms were disabled, allowing the function to evaluate the full model space.

The integrated (d) component of the ARIMA model addresses non-stationarity through differencing; the auto.arima() function automatically determined the appropriate degree of differencing by minimizing the AICc. No additional transformation (e.g., logarithmic or Box-Cox) was applied as the variance of the age-standardized rate series appeared stable across the observation period. Forecast intervals were derived from the model’s conditional variance, reflecting parameter uncertainty and residual error. We selected ARIMA because it is widely validated for epidemiological time-series forecasting and requires fewer assumptions about demographic structures compared with age-period-cohort models, which is advantageous given the relatively short observation period (1990–2023).

### 2.6. Ethics statement

This study used publicly available, aggregated population-level data from the GBD Study 2023. No individual patient data were accessed, and no human subjects were directly involved. Therefore, ethical approval and informed consent were not required for this secondary analysis.

## 3. Results

### 3.1. Disease burden in 2023

In 2023, Asia recorded approximately 1.87 million incident PUD cases (ASIR: 39.34 per 100,000; 95% UI: 24.86–57.89) and 4.32 million prevalent cases (ASPR: 90.58 per 100,000; 95% UI: 61.44–129.39). The disease caused 131,011 deaths (ASDR: 2.85 per 100,000; 95% UI: 1.86–4.32) and 3.46 million DALYs (age-standardized rate: 73.1 per 100,000; 95% UI: 47.97–111.68). Notably, premature mortality (YLLs) accounted for more than 90% of total DALYs, whereas YLDs contributed only approximately 6%, indicating that the overwhelming burden of PUD in Asia stems from fatal complications rather than long-term disability. Figure 1 presents the disease burden rates of PUD in Asia across age groups. Table 1 shows the disease burden of PUD in Asia by country and territory.

**Table 1:** Disease burden of PUD across Asia in 2023.

Location/Indicator	Incidence cases	ASIR	Prevalence cases	ASPR	Deaths cases	ASDR
Afghanistan	11,556 (9511–13,528)	57.05 (49.85–65.48)	27,283 (23,198–32,226)	129.37 (112.56–147.39)	461 (219–799)	3.65 (1.37–6.21)
Armenia	1599 (1388–1858)	35.88 (31.14–40.88)	3625 (3196–4116)	81.24 (71.75–92.70)	280 (249–318)	5.55 (4.94–6.31)
Azerbaijan	2889 (2483–3281)	24.95 (21.35–28.16)	6440 (5544–7516)	55.10 (47.47–64.21)	240 (149–380)	2.18 (1.40–3.34)
Bahrain	429 (343–517)	35.32 (30.17–40.27)	993 (797–1224)	75.47 (64.62–89.30)	19 (12–33)	3.72 (2.12–7.09)
Bangladesh	44,340 (34,561–54,580)	25.40 (20.13–30.57)	107,144 (85,549–135,022)	59.96 (48.44–74.64)	2327 (1445–3593)	1.87 (1.21–2.76)
Bhutan	320 (270–366)	44.28 (38.20–50.42)	683 (587–794)	92.42 (79.82–106.41)	59 (23–152)	9.26 (3.80–23.31)
Brunei Darussalam	150 (126–178)	32.64 (27.80–38.00)	350 (294–423)	73.70 (62.96–87.70)	6 (4–9)	1.69 (1.21–2.36)
Cambodia	14,752 (12,676–17,010)	99.48 (86.84–114.40)	36,444 (31,731–41,337)	240.77 (209.37–274.32)	1423 (766–2271)	11.35 (6.19–17.54)
China	661,788 (550,310–802,414)	32.21 (26.97–37.91)	1559,208 (1317,004−1831,326)	75.17 (64.43–88.04)	34,767 (27,852–43,872)	1.61 (1.28–2.05)
Democratic People’s Republic of Korea	23,764 (19,976–28,767)	71.12 (60.39–84.01)	59,237 (49,443–68,482)	175.46 (149.51–201.54)	1037 (482–1842)	3.50 (1.61–6.26)
Georgia	1475 (1277–1692)	30.15 (25.92–34.34)	3354 (2952–3782)	68.68 (60.77–78.22)	415 (352–490)	6.89 (5.80–8.13)
India	577,760 (485,113–664,131)	41.64 (35.58–47.79)	1279,221 (1107,131–1500,848)	89.98 (78.33–105.78)	47,576 (33,043–72,017)	4.17 (2.86–6.34)
Indonesia	59,210 (46,722–71,861)	20.54 (16.61–24.40)	137,525 (107,848–175,405)	46.33 (37.18–58.36)	413 (264–619)	0.17 (0.11–0.25)
Iran (Islamic Republic of)	20,998 (17,406–24,816)	23.93 (20.12–27.85)	48,509 (40,441–58,268)	53.96 (45.54–65.89)	1290 (808–2024)	2.01 (1.26–3.20)
Iraq	7758 (6103–9455)	20.01 (16.31–23.75)	18,067 (14,465–22,691)	45.87 (37.85–56.34)	229 (131–362)	1.04 (0.60–1.60)
Japan	87,375 (74,301–101,634)	41.65 (34.56–48.82)	208,964 (180,575–242,247)	101.06 (86.10–121.78)	4052 (3160–4769)	0.79 (0.65–0.90)
Jordan	2414 (1947–2874)	21.26 (17.99–24.80)	5428 (4347–6736)	46.25 (38.42–55.89)	58 (42–86)	0.84 (0.59–1.24)
Kazakhstan	5866 (4955–6739)	27.77 (23.61–31.77)	13,445 (11,651–15,486)	63.58 (55.20–73.43)	825 (737–920)	4.23 (3.83–4.67)
Kuwait	1129 (871–1416)	22.87 (18.85–27.59)	2712 (2117–3484)	52.65 (42.85–65.41)	11 (9–13)	0.62 (0.49–0.75)
Kyrgyzstan	1494 (1257–1727)	22.42 (19.04–25.77)	3389 (2883–3983)	50.65 (43.60–59.54)	99 (84–115)	1.99 (1.71–2.31)
Lao People’s Democratic Republic	5376 (4611–6391)	90.96 (78.98–107.63)	13,206 (11,623–14,989)	219.51 (190.41–251.75)	450 (273–667)	10.14 (6.33–14.97)
Lebanon	1686 (1410–1942)	27.68 (23.16–31.99)	3832 (3226–4531)	62.82 (53.19–74.74)	68 (46–96)	1.08 (0.74–1.51)
Malaysia	15,055 (13,117–17,443)	44.59 (39.21–51.50)	33,971 (29,602–39,392)	99.10 (86.76–113.83)	930 (681–1291)	3.29 (2.38–4.62)
Maldives	161 (122–198)	29.38 (23.99–34.98)	384 (299–492)	67.30 (54.70–83.31)	4 (2–6)	1.66 (0.95–3.09)
Mauritius	480 (418–540)	29.79 (25.63–33.62)	1088 (947–1268)	67.22 (57.75–78.85)	26 (23–31)	1.51 (1.29–1.78)
Mongolia	1423 (1198–1645)	49.48 (42.97–57.04)	3312 (2885–3755)	113.65 (99.13–128.80)	132 (92–184)	5.68 (3.87–7.92)
Myanmar	30,266 (25,812–34,640)	54.47 (47.01–62.07)	71,837 (62,962–81,066)	127.78 (112.56–144.04)	5871 (3178–9693)	11.51 (6.43–18.67)
Nepal	9122 (6827–11,285)	29.44 (22.39–36.18)	21,495 (16,691–27,758)	67.91 (53.42–87.84)	2204 (1309–4368)	9.14 (5.44–17.76)
Oman	1142 (891–1442)	26.61 (22.51–31.06)	2712 (2073–3527)	59.77 (49.95–72.83)	26 (15–47)	1.51 (0.85–2.74)
Pakistan	60,923 (48,941–72,843)	31.80 (26.81–37.15)	135,002 (109,951–167,269)	68.76 (57.61–84.00)	11,672 (7325–18,933)	8.54 (5.52–12.99)
Palestine	1060 (829–1294)	24.46 (20.26–28.95)	2496 (1986–3184)	55.97 (46.34–68.32)	15 (10–22)	0.65 (0.45–0.98)
Philippines	76,219 (63,593–89,999)	76.75 (65.17–90.55)	179,927 (156,074–211,885)	177.66 (153.32–208.79)	6618 (5228–8424)	8.61 (6.74–10.90)
Qatar	781 (569–1035)	27.41 (22.98–32.23)	1899 (1373–2545)	61.80 (51.10–75.38)	4 (2–7)	0.85 (0.41–1.85)
Republic of Korea	22,074 (18,025–25,851)	30.91 (24.83–36.91)	53,169 (44,507–65,709)	75.26 (60.36–93.84)	744 (516–926)	0.76 (0.53–0.95)
Saudi Arabia	6759 (5178–8422)	23.43 (19.23–27.24)	15,844 (12,052–20,502)	52.01 (43.50–63.38)	203 (139–282)	1.97 (1.40–2.70)
Seychelles	70 (60–82)	56.93 (48.99–66.68)	168 (145–191)	134.38 (117.13–154.00)	7 (5–10)	5.96 (4.08–8.61)
Singapore	2192 (1735–2623)	29.71 (23.50–35.86)	5431 (4403–6857)	73.47 (58.80–91.33)	56 (46–67)	0.62 (0.51–0.73)
Sri Lanka	6025 (4789–7157)	23.90 (18.66–28.90)	14,202 (11,395–17,555)	56.15 (44.56–70.16)	119 (76–183)	0.41 (0.26–0.63)
Syrian Arab Republic	2995 (2378–3565)	17.40 (14.21–20.54)	6774 (5441–8405)	38.77 (31.58–47.51)	100 (61–159)	0.97 (0.62–1.57)
Taiwan (Province of China)	18,815 (16,020–22,243)	55.46 (45.77–65.51)	46,254 (39,709–55,282)	137.39 (114.34–167.86)	822 (713–965)	1.81 (1.57–2.12)
Tajikistan	2304 (1907–2662)	27.39 (23.61–30.98)	5212 (4467–6171)	61.06 (52.86–71.01)	198 (137–263)	3.50 (2.43–4.81)
Thailand	26,285 (22,759–30,069)	29.45 (24.68–34.29)	59,280 (50,615–69,902)	67.15 (56.03–81.34)	1455 (1109–1872)	1.27 (0.98–1.63)
Timor-Leste	860 (720–998)	84.29 (72.91–99.55)	2058 (1758–2369)	198.41 (168.07–225.48)	128 (72–204)	15.46 (8.73–24.89)
Turkmenistan	1524 (1307–1736)	28.32 (24.52–32.14)	3579 (3106–4110)	66.25 (57.86–75.83)	143 (115–175)	3.11 (2.57–3.76)
United Arab Emirates	2610 (1986–3280)	25.73 (21.76–29.97)	6143 (4640–7799)	56.96 (47.65–69.24)	37 (24–57)	2.36 (1.38–4.35)
Uzbekistan	10,372 (8891–12,002)	31.65 (27.56–36.05)	23,988 (20,689–27,482)	72.70 (62.60–82.80)	805 (716–900)	3.02 (2.71–3.35)
Viet Nam	28,469 (22,691–34,553)	25.77 (20.54–30.78)	65,704 (52,319–81,147)	59.16 (47.25–74.06)	1786 (774–3864)	1.69 (0.74–3.65)
Yemen	9679 (7975–11,169)	43.73 (37.91–50.66)	22,100 (18,948–26,363)	96.82 (85.62–110.54)	802 (396–1499)	6.72 (3.16–12.43)

Location/Indicator	DALYs years	Age-standardized DALYs rate	YLLs years	Age-standardized YLLs rate	YLDs years	Age-standardized YLDs rate
Afghanistan	19,216 (11,561–31,825)	97.87 (47.57–164.30)	17,815 (10,051–30,663)	91.41 (42.00–156.82)	1401 (945–1997)	6.47 (4.35–9.14)
Armenia	5920 (5304–6694)	128.34 (114.62–145.28)	5731 (5084–6529)	124.11 (109.71–141.07)	189 (131–263)	4.23 (2.88–5.90)
Azerbaijan	7637 (4804–12,529)	64.50 (40.73–104.38)	7280 (4387–12,240)	61.44 (37.04–101.22)	356 (248–491)	3.06 (2.12–4.25)
Bahrain	524 (342–828)	60.84 (39.03–108.98)	476 (284–784)	57.17 (34.96–104.71)	49 (31–70)	3.66 (2.45–5.22)
Bangladesh	73,426 (45,854–113,024)	50.28 (32.26–76.59)	67,742 (40,294–107,242)	47.10 (28.89–73.42)	5684 (3388–8544)	3.19 (1.92–4.76)
Bhutan	1536 (570–4259)	220.74 (83.93–590.17)	1500 (533–4225)	215.79 (79.07–585.60)	36 (24–53)	4.95 (3.31–7.19)
Brunei Darussalam	176 (128–253)	39.79 (29.19–55.76)	157 (109–231)	35.85 (25.15–51.52)	19 (12–27)	3.94 (2.65–5.70)
Cambodia	37,096 (19,382–63,875)	258.97 (138.34–427.93)	35,229 (16,942–61,974)	246.63 (123.23–413.75)	1867 (1262–2666)	12.34 (8.32–17.56)
China	737,096 (595,993–910,175)	34.65 (28.13–42.97)	665,310 (514,020–833,326)	31.17 (24.15–39.87)	71,786 (49,112–100,929)	3.47 (2.36–4.92)
Democratic People’s Republic of Korea	28,761 (15,167–49,398)	88.55 (46.71–152.24)	25,923 (12,367–46,935)	80.13 (37.91–143.38)	2838 (1969–4095)	8.43 (5.84–12.03)
Georgia	8926 (7390–10,529)	162.31 (133.24–192.27)	8726 (7181–10,347)	158.23 (129.18–187.80)	200 (140–275)	4.08 (2.81–5.62)
India	1343,966 (950,472–1990,552)	102.57 (72.17–152.10)	1275,230 (885,948–1923,343)	97.76 (67.53–147.44)	68,735 (46,508–96,431)	4.81 (3.28–6.76)
Indonesia	21,505 (15,495–29,875)	7.53 (5.40–10.30)	14,238 (8879–21,764)	5.07 (3.17–7.68)	7267 (4795–10,497)	2.47 (1.63–3.59)
Iran (Islamic Republic of)	31,246 (20,057–47,390)	40.77 (26.17–64.21)	28,806 (17,586–45,405)	38.08 (23.50–62.26)	2440 (1657–3502)	2.70 (1.82–3.87)
Iraq	8169 (4849–13,040)	26.26 (15.74–39.88)	7255 (3930–12,157)	23.94 (13.53–37.69)	914 (580–1309)	2.32 (1.55–3.29)
Japan	66,026 (55,518–75,807)	19.80 (17.02–22.39)	54,475 (44,931–62,564)	14.21 (12.26–16.04)	11,551 (7990–15,897)	5.59 (3.80–7.86)
Jordan	1762 (1289–2475)	18.87 (14.05–26.50)	1483 (1009–2185)	16.51 (11.61–24.40)	280 (185–399)	2.36 (1.57–3.31)
Kazakhstan	22,365 (19,896–25,039)	105.73 (94.32–117.79)	21,647 (19,283–24,314)	102.32 (91.08–114.82)	718 (493–1041)	3.41 (2.33–4.92)
Kuwait	425 (349–521)	13.17 (11.16–15.92)	294 (239–360)	10.62 (8.72–13.03)	131 (83–189)	2.55 (1.64–3.60)
Kyrgyzstan	3179 (2696–3670)	53.24 (45.62–61.33)	2999 (2531–3514)	50.52 (42.95–58.91)	180 (118–258)	2.72 (1.82–3.87)
Lao People’s Democratic Republic	12,044 (7651–18,031)	227.42 (142.20–338.56)	11,383 (6817–17,327)	216.39 (131.84–326.15)	661 (454–926)	11.03 (7.55–15.64)
Lebanon	1612 (1194–2210)	26.76 (19.90–36.74)	1428 (1026–2017)	23.73 (17.01–33.78)	184 (123–266)	3.02 (2.04–4.34)
Malaysia	21,608 (15,988–29,172)	67.84 (50.04–92.13)	19,978 (14,444–27,528)	63.07 (46.09–87.45)	1630 (1072–2346)	4.77 (3.14–6.89)
Maldives	97 (67–139)	30.13 (19.38–48.47)	77 (48–115)	26.70 (15.83–44.78)	19 (12–29)	3.42 (2.19–5.06)
Mauritius	633 (540–733)	35.56 (30.42–41.17)	579 (491–681)	32.24 (27.41–38.06)	54 (36–78)	3.32 (2.29–4.78)
Mongolia	4186 (2936–5723)	146.61 (102.72–200.47)	4019 (2792–5557)	140.81 (97.87–195.26)	168 (113–243)	5.81 (3.98–8.41)
Myanmar	208,126 (110,208–349,048)	372.30 (200.45–617.80)	204,411 (106,399–345,775)	365.68 (193.72–611.97)	3715 (2538–5263)	6.62 (4.52–9.32)
Nepal	60,706 (35,803–124,018)	222.24 (131.81–449.98)	59,552 (34,900–122,831)	218.58 (128.56–446.22)	1154 (740–1739)	3.66 (2.36–5.44)
Oman	896 (575–1490)	31.64 (19.44–54.68)	758 (414–1355)	28.57 (16.42–51.23)	138 (82–218)	3.07 (2.00–4.39)
Pakistan	365,756 (224,001–642,047)	220.72 (136.49–378.41)	358,196 (216,002–636,496)	216.89 (133.01–374.42)	7560 (4951–10,860)	3.83 (2.58–5.45)
Palestine	486 (355–672)	15.36 (11.33–22.08)	357 (245–514)	12.49 (8.70–18.24)	129 (79–186)	2.87 (1.83–4.16)
Philippines	186,551 (146,222–244,927)	204.21 (161.10–262.24)	177,813 (137,759–234,698)	195.57 (153.32–254.96)	8738 (5983–12,122)	8.64 (5.91–12.01)
Qatar	207 (143–297)	15.37 (9.17–27.11)	116 (64–191)	12.43 (6.39–24.78)	90 (53–136)	2.94 (1.91–4.16)
Republic of Korea	14,215 (10,056–17,573)	15.88 (11.57–19.68)	11,367 (7439–14,210)	11.95 (8.04–14.76)	2847 (1909–3963)	3.93 (2.59–5.66)
Saudi Arabia	6987 (5004–9607)	38.55 (27.69–52.00)	6206 (4314–8760)	36.00 (24.83–49.72)	782 (466–1208)	2.55 (1.68–3.70)
Seychelles	178 (122–263)	142.36 (98.59–209.41)	170 (115–254)	135.89 (93.17–202.28)	8 (6–12)	6.47 (4.40–9.16)
Singapore	1224 (1054–1423)	14.29 (12.24–16.80)	945 (817–1107)	10.56 (9.11–12.37)	279 (179–406)	3.73 (2.41–5.49)
Sri Lanka	2947 (2119–3886)	10.39 (7.50–13.49)	2221 (1445–3228)	7.53 (4.87–10.90)	726 (462–1076)	2.86 (1.78–4.27)
Syrian Arab Republic	2605 (1736–4055)	18.72 (12.42–29.57)	2249 (1394–3629)	16.69 (10.51–27.19)	356 (221–499)	2.04 (1.25–2.90)
Taiwan (Province of China)	17,089 (14,908–20,000)	42.82 (37.08–50.07)	14,836 (12,908–17,575)	36.15 (30.96–43.04)	2253 (1547–3109)	6.67 (4.48–9.48)
Tajikistan	6564 (4684–8611)	90.47 (63.93–119.28)	6281 (4370–8396)	87.11 (60.52–116.50)	283 (190–399)	3.36 (2.27–4.71)
Thailand	31,775 (25,773–38,597)	30.55 (24.68–37.51)	28,715 (23,119–35,512)	27.12 (21.65–33.65)	3060 (2032–4373)	3.44 (2.25–4.85)
Timor-Leste	3239 (1849–4975)	342.57 (195.71–534.09)	3136 (1751–4847)	332.73 (186.86–522.50)	102 (68–145)	9.84 (6.63–13.94)
Turkmenistan	5084 (4046–6319)	99.03 (79.84–121.76)	4898 (3850–6074)	95.54 (76.51–117.77)	186 (128–274)	3.49 (2.40–5.10)
United Arab Emirates	1308 (937–1902)	34.68 (22.00–57.69)	1013 (638–1598)	31.95 (19.59–55.89)	295 (178–439)	2.73 (1.75–3.86)
Uzbekistan	26,189 (23,559–29,406)	84.31 (76.07–93.95)	24,880 (22,106–28,107)	80.33 (71.17–90.21)	1309 (868–1857)	3.98 (2.64–5.60)
Viet Nam	32,882 (16,634–67,756)	30.74 (15.52–63.49)	29,591 (13,124–63,443)	27.78 (12.22–59.57)	3291 (2158–4853)	2.96 (1.94–4.38)
Yemen	23,001 (12,122–41,224)	136.00 (69.10–247.55)	21,772 (10,756–39,651)	130.78 (64.67–242.64)	1230 (827–1751)	5.22 (3.61–7.35)

**Figure 1.**
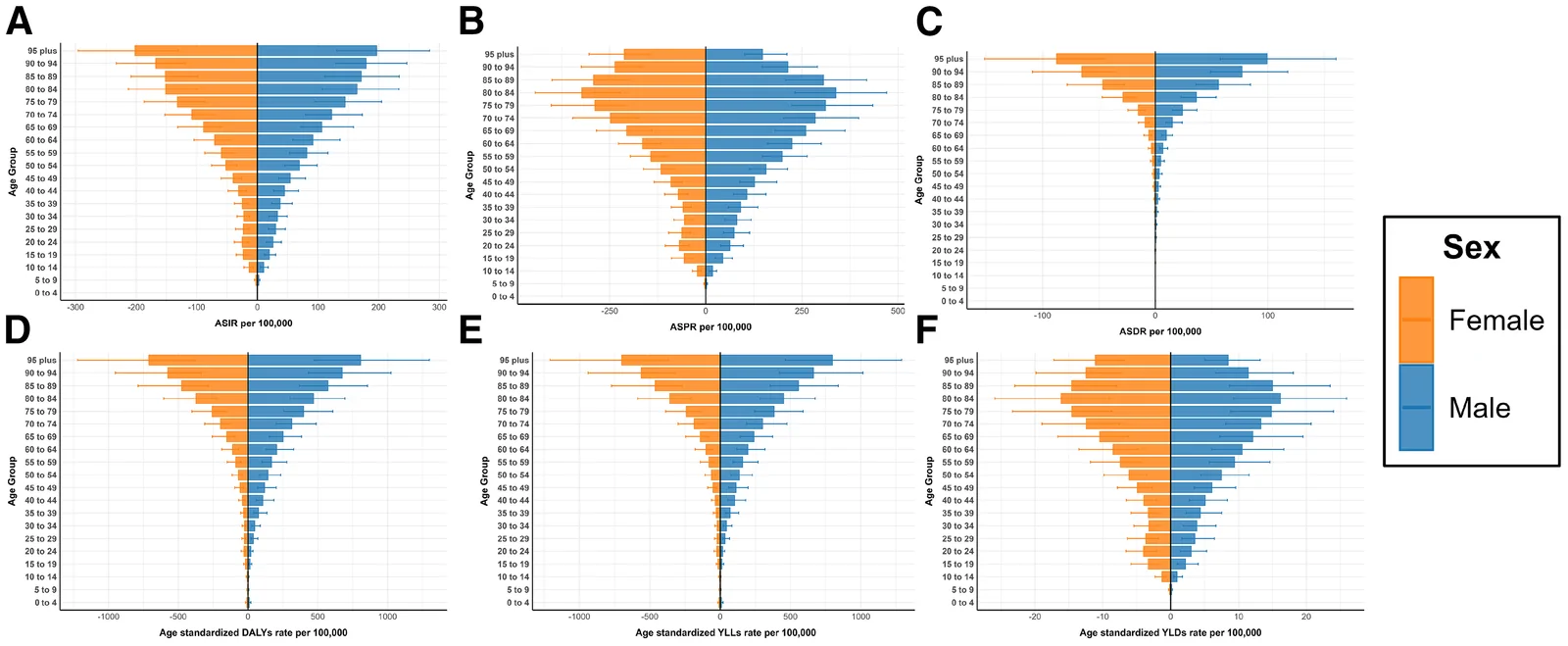
Disease burden of different age groups in PUD across Asian countries and territories. (A) ASIR of PUD in different age groups. (B) ASPR of PUD in different age groups. (C) ASDR of PUD in different age groups. (D) Age-standardized DALYs rates of PUD in different age groups. (E) Age-standardized YLLs rates of PUD in different age groups. (F) Age-standardized YLDs rates of PUD in different age groups. ASDR = age-standardized death rate, ASIR = age-standardized incidence rate, ASPR = age-standardized prevalence rate, PUD = peptic ulcer disease, YLDs = years lived with disability.

### 3.2. Joinpoint regression

From 1990 to 2023, Joinpoint regression revealed significant declining trends across all age-standardized metrics in Asia (all *P* < .05). The ASIR declined with an AAPC of −1.32% (95% CI: −1.34–− 1.30), the ASPR with −1.41% (95% CI: −1.43–−1.39), the ASDR with −2.65% (95% CI: −2.72–− 2.59), and the age-standardized DALY rate with − 3.18% (95% CI: −3.24–− 3.13). Similar declines were observed for YLLs (AAPC: −3.27%; 95% CI: −3.33 to − 3.21) and YLDs (AAPC: −1.52%; 95% CI: −1.53–−1.50). Figure 2 illustrates the secular trend of PUD in Asia over the past 33 years. Table 2 presents the country-specific AAPC values for PUD in Asia during the same period.

**Table 2:** Temporal trends (AAPC value) in the disease burden of PUD in Asian Countries and regions, 1990 to 2023.

Countries and regions/type	ASIR		ASPR		ASDR	
	AAPC	*P* value	AAPC	*P* value	AAPC	*P* value
Afghanistan	−0.8 (−0.81–−0.78)	<.05	−0.99 (−1.01–−0.97)	<.05	−2.64 (−2.7–−2.57)	<.05
Armenia	0.18 (0.16–0.19)	<.05	0.11 (0.1–0.13)	<.05	−1.36 (−1.55–−1.19)	<.05
Azerbaijan	−1.03 (−1.06–−1.01)	<.05	−1.19 (−1.22–−1.17)	<.05	−3.02 (−3.14–−2.91)	<.05
Bahrain	−1.22 (−1.25–−1.19)	<.05	−1.2 (−1.23–−1.17)	<.05	−4.38 (−4.47–−4.27)	<.05
Bangladesh	−3.18 (−3.22–−3.13)	<.05	−3.12 (−3.18–−3.07)	<.05	−6.93 (−7.07–−6.8)	<.05
Bhutan	−2.1 (−2.12–−2.09)	<.05	−2.41 (−2.43–−2.4)	<.05	−3.22 (−3.28–−3.14)	<.05
Brunei Darussalam	−2.18 (−2.21–−2.16)	<.05	−2.48 (−2.5–−2.45)	<.05	−4.49 (−4.54–−4.44)	<.05
Cambodia	−1.46 (−1.47–−1.45)	<.05	−1.8 (−1.81–−1.79)	<.05	−2.02 (−2.1–−1.93)	<.05
China	−2.4 (−2.43–−2.37)	<.05	−2.47 (−2.5–−2.44)	<.05	−4.26 (−4.35–−4.16)	<.05
Democratic People’s Republic of Korea	−0.64 (−0.65–−0.63)	<.05	−0.83 (−0.85–−0.82)	<.05	−2.25 (−2.29–−2.22)	<.05
Georgia	−0.34 (−0.36–−0.32)	<.05	−0.43 (−0.45–−0.42)	<.05	1.62 (1.25–1.97)	<.05
India	−2.62 (−2.64–−2.6)	<.05	−2.7 (−2.72–−2.68)	<.05	−4.06 (−4.18–−3.95)	<.05
Indonesia	−0.76 (−0.79–−0.73)	<.05	−0.74 (−0.77–−0.7)	<.05	−3.67 (−3.78–−3.56)	<.05
Iran (Islamic Republic of)	−1.78 (−1.81–−1.75)	<.05	−1.76 (−1.79–−1.73)	<.05	−4.12 (−4.52–−3.76)	<.05
Iraq	−0.89 (−0.92–−0.87)	<.05	−0.86 (−0.89–−0.83)	<.05	−4.3 (−4.48–−4.07)	<.05
Japan	−0.49 (−0.53–−0.46)	<.05	−0.42 (−0.45–−0.39)	<.05	−3.7 (−3.87–−3.54)	<.05
Jordan	−1.48 (−1.51–−1.46)	<.05	−1.41 (−1.43–−1.38)	<.05	−5.62 (−5.74–−5.47)	<.05
Kazakhstan	−0.17 (−0.18–−0.15)	<.05	−0.2 (−0.21–−0.18)	<.05	1.09 (0.71–1.48)	<.05
Kuwait	−0.72 (−0.75–−0.69)	<.05	−0.7 (−0.72–−0.67)	<.05	−4.28 (−4.65–−3.92)	<.05
Kyrgyzstan	−1.28 (−1.3–−1.27)	<.05	−1.39 (−1.41–−1.37)	<.05	−2.53 (−2.83–−2.28)	<.05
Lao People’s Democratic Republic	−1.62 (−1.63–−1.61)	<.05	−2 (−2.01–−1.99)	<.05	−2.13 (−2.15–−2.11)	<.05
Lebanon	−0.72 (−0.73–−0.7)	<.05	−0.68 (−0.7–−0.66)	<.05	−5.43 (−5.66–−5.19)	<.05
Malaysia	−0.32 (−0.35–−0.3)	<.05	−0.31 (−0.32–−0.29)	<.05	−3.62 (−3.77–−3.45)	<.05
Maldives	−1.22 (−1.26–−1.19)	<.05	−1.13 (−1.17–−1.1)	<.05	−3.69 (−3.76–−3.61)	<.05
Mauritius	−2.14 (−2.16–−2.13)	<.05	−2.42 (−2.43–−2.4)	<.05	−5.14 (−5.39–−4.91)	<.05
Mongolia	−0.79 (−0.8–−0.77)	<.05	−0.76 (−0.78–−0.74)	<.05	−1.41 (−1.57–−1.26)	<.05
Myanmar	−2.2 (−2.21–−2.18)	<.05	−2.65 (−2.67–−2.64)	<.05	−3.33 (−3.44–−3.18)	<.05
Nepal	−0.08 (−0.12–−0.04)	<.05	0.14 (0.1–0.18)	<.05	−3.98 (−4.12–−3.76)	<.05
Oman	−1.04 (−1.06–−1.03)	<.05	−1.09 (−1.11–−1.07)	<.05	−4.05 (−4.24–−3.88)	<.05
Pakistan	−0.94 (−0.97–−0.91)	<.05	−0.95 (−0.98–−0.91)	<.05	−2.5 (−2.58–−2.39)	<.05
Palestine	−0.66 (−0.69–−0.63)	<.05	−0.68 (−0.72–−0.64)	<.05	−4.75 (−4.91–−4.61)	<.05
Philippines	−2.11 (−2.12–−2.09)	<.05	−2.24 (−2.25–−2.22)	<.05	−2.04 (−2.1–−1.98)	<.05
Qatar	0.28 (0.25–0.3)	<.05	0.36 (0.33–0.39)	<.05	−7.82 (−8.22–−7.54)	<.05
Republic of Korea	−2 (−2.04–−1.98)	<.05	−2.06 (−2.09–−2.03)	<.05	−7.09 (−7.22–−7)	<.05
Saudi Arabia	−0.93 (−0.96–−0.91)	<.05	−0.85 (−0.87–−0.83)	<.05	−2.15 (−2.36–−1.97)	<.05
Seychelles	−1.01 (−1.03–−0.99)	<.05	−1.26 (−1.28–−1.25)	<.05	−3.58 (−3.74–−3.41)	<.05
Singapore	−1.52 (−1.55–−1.49)	<.05	−1.57 (−1.6–−1.54)	<.05	−5.27 (−5.5–−5.07)	<.05
Sri Lanka	−0.34 (−0.37–−0.31)	<.05	−0.23 (−0.27–−0.2)	<.05	−5.29 (−5.53–−5.07)	<.05
Syrian Arab Republic	−0.17 (−0.2–−0.15)	<.05	−0.03 (−0.06–0)	<.05	−3.72 (−3.8–−3.63)	<.05
Taiwan (Province of China)	−2.15 (−2.45–−1.95)	<.05	−2.55 (−3–−2.25)	<.05	−4.91 (−5.13–−4.77)	<.05
Tajikistan	−1.13 (−1.15–−1.1)	<.05	−1.4 (−1.43–−1.36)	<.05	−1.68 (−1.88–−1.45)	<.05
Thailand	−0.12 (−0.14–−0.1)	<.05	−0.08 (−0.1–−0.06)	<.05	6.32 (6.07–6.54)	<.05
Timor-Leste	−1.08 (−1.09–−1.07)	<.05	−1.37 (−1.39–−1.36)	<.05	−1.77 (−1.83–−1.71)	<.05
Turkmenistan	−0.08 (−0.1–−0.05)	<.05	−0.02 (−0.05–0)	0.1	−0.11 (−0.68–0.31)	.4
United Arab Emirates	−1.09 (−1.11–−1.07)	<.05	−1.1 (−1.12–−1.08)	<.05	−4.37 (−4.49–−4.25)	<.05
Uzbekistan	−0.27 (−0.29–−0.25)	<.05	−0.36 (−0.39–−0.33)	<.05	−1.94 (−2.13–−1.73)	<.05
Viet Nam	−0.4 (−0.43–−0.37)	<.05	−0.25 (−0.28–−0.22)	<.05	−2.63 (−2.72–−2.45)	<.05
Yemen	−0.86 (−0.87–−0.86)	<.05	−1.02 (−1.02–−1.01)	<.05	−3.35 (−3.48–−3.24)	<.05

Countries and regions/ Type	Age-standardized DALYs rate		Age-standardized YLLs rate		Age-standardized YLDs rate	
	AAPC	*P* Value	AAPC	*P* Value	AAPC	P.Value
Afghanistan	−2.55 (−2.71–−2.43)	<.05	−2.63 (−2.79–−2.49)	<.05	−1.05 (−1.07–−1.02)	<0.05
Armenia	−1.54 (−1.71–−1.28)	<.05	−1.58 (−1.76–−1.32)	<.05	0.1 (0.08–0.12)	<0.05
Azerbaijan	−3.05 (−3.17–−2.92)	<.05	−3.11 (−3.24–−2.98)	<.05	−1.15 (−1.17–−1.13)	<0.05
Bahrain	−4.85 (−4.94–−4.74)	<.05	−4.97 (−5.07–−4.86)	<.05	−1.38 (−1.41–−1.34)	<0.05
Bangladesh	−6.99 (−7.11–−6.87)	<.05	−7.13 (−7.25–−6.99)	<.05	−3.14 (−3.19–−3.1)	<0.05
Bhutan	−3.69 (−3.76–−3.61)	<.05	−3.72 (−3.78–−3.64)	<.05	−2.47 (−2.48–−2.45)	<0.05
Brunei Darussalam	−4.47 (−4.53–−4.42)	<.05	−4.61 (−4.69–−4.55)	<.05	−2.32 (−2.35–−2.29)	<0.05
Cambodia	−2.42 (−2.5–−2.31)	<.05	−2.44 (−2.53–−2.32)	<.05	−1.92 (−1.94–−1.91)	<0.05
China	−4.71 (−4.78–−4.62)	<.05	−4.86 (−4.95–−4.77)	<.05	−2.69 (−2.72–−2.66)	<0.05
Democratic People’s Republic of Korea	−2.16 (−2.2–−2.13)	<.05	−2.26 (−2.31–−2.23)	<.05	−0.99 (−1–−0.97)	<0.05
Georgia	0.85 (0.53–1.16)	<.05	0.88 (0.54–1.21)	<.05	−0.29 (−0.32–−0.26)	<0.05
India	−4.5 (−4.62–−4.4)	<.05	−4.56 (−4.68–−4.46)	<.05	−2.72 (−2.74–−2.7)	<0.05
Indonesia	−3.03 (−3.21–−2.92)	<.05	−3.67 (−3.8–−3.54)	<.05	−0.84 (−0.87–−0.81)	<0.05
Iran (Islamic Republic of)	−4.91 (−5.29–−4.65)	<.05	−5.04 (−5.45–−4.77)	<.05	−1.92 (−1.95–−1.9)	<0.05
Iraq	−4.31 (−4.5–−4.15)	<.05	−4.5 (−4.7–−4.32)	<.05	−1 (−1.03–−0.97)	<0.05
Japan	−2.94 (−3.08–−2.78)	<.05	−3.6 (−3.72–−3.49)	<.05	−0.42 (−0.45–−0.4)	<0.05
Jordan	−5.46 (−5.58–−5.32)	<.05	−5.74 (−5.87–−5.6)	<.05	−1.45 (−1.48–−1.41)	<0.05
Kazakhstan	0.47 (0.19–0.78)	<.05	0.5 (0.21–0.82)	<.05	−0.26 (−0.28–−0.23)	<0.05
Kuwait	−4.16 (−4.51–−3.78)	<.05	−4.62 (−5–−4.24)	<.05	−0.79 (−0.82–−0.76)	<0.05
Kyrgyzstan	−3.16 (−3.43–−2.91)	<.05	−3.23 (−3.51–−2.97)	<.05	−1.38 (−1.41–−1.36)	<0.05
Lao People’s Democratic Republic	−2.48 (−2.5–−2.46)	<.05	−2.5 (−2.52–−2.48)	<.05	−2.07 (−2.09–−2.06)	<0.05
Lebanon	−5.03 (−5.2–−4.85)	<.05	−5.31 (−5.51–−5.11)	<.05	−0.96 (−0.99–−0.93)	<0.05
Malaysia	−3.69 (−3.84–−3.48)	<.05	−3.88 (−4.09–−3.7)	<.05	−0.39 (−0.41–−0.37)	<0.05
Maldives	−4.42 (−4.49–−4.32)	<.05	−4.64 (−4.72–−4.53)	<.05	−1.31 (−1.34–−1.27)	<0.05
Mauritius	−5.21 (−5.4–−4.94)	<.05	−5.39 (−5.59–−5.1)	<.05	−2.44 (−2.46–−2.41)	<0.05
Mongolia	−1.41 (−1.57–−1.24)	<.05	−1.43 (−1.6–−1.26)	<.05	−0.9 (−0.92–−0.87)	<0.05
Myanmar	−3.32 (−3.44–−3.11)	<.05	−3.33 (−3.45–−3.13)	<.05	−2.69 (−2.71–−2.67)	<0.05
Nepal	−4.48 (−4.67–−4.15)	<.05	−4.52 (−4.71–−4.18)	<.05	−0.21 (−0.25–−0.17)	<0.05
Oman	−4.34 (−4.62–−4.14)	<.05	−4.54 (−4.85–−4.33)	<.05	−1.15 (−1.18–−1.12)	<0.05
Pakistan	−2.71 (−2.82–−2.55)	<.05	−2.73 (−2.85–−2.57)	<.05	−0.95 (−0.98–−0.92)	<0.05
Palestine	−4.53 (−4.76–−4.37)	<.05	−4.97 (−5.14–−4.83)	<.05	−0.89 (−0.92–−0.86)	<0.05
Philippines	−2.13 (−2.2–−2.08)	<.05	−2.13 (−2.19–−2.07)	<.05	−2.38 (−2.39–−2.36)	<0.05
Qatar	−7.74 (−8.04–−7.53)	<.05	−8.29 (−8.64–−8.05)	<.05	0.1 (0.07–0.14)	<0.05
Republic of Korea	−7.03 (−7.11–−6.97)	<.05	−7.71 (−7.8–−7.64)	<.05	−2.03 (−2.06–−2)	<0.05
Saudi Arabia	−2.63 (−2.79–−2.48)	<.05	−2.72 (−2.88–−2.56)	<.05	−1.05 (−1.07–−1.02)	<0.05
Seychelles	−3.68 (−3.79–−3.57)	<.05	−3.75 (−3.87–−3.64)	<.05	−1.38 (−1.4–−1.35)	<0.05
Singapore	−5.04 (−5.23–−4.86)	<.05	−5.63 (−5.83–−5.46)	<.05	−1.58 (−1.61–−1.54)	<0.05
Sri Lanka	−4.84 (−5.04–−4.66)	<.05	−5.63 (−5.93–−5.38)	<.05	−0.4 (−0.44–−0.36)	<0.05
Syrian Arab Republic	−3.98 (−4.07–−3.88)	<.05	−4.23 (−4.34–−4.11)	<.05	−0.23 (−0.27–−0.19)	<0.05
Taiwan (Province of China)	−4.44 (−4.59–−4.3)	<.05	−4.66 (−4.88–−4.52)	<.05	−2.53 (−2.96–−2.23)	<0.05
Tajikistan	−2.11 (−2.38–−1.77)	<.05	−2.14 (−2.41–−1.8)	<.05	−1.37 (−1.4–−1.33)	<0.05
Thailand	4.12 (3.91–4.31)	<.05	5.73 (5.5–6.02)	<.05	−0.15 (−0.18–−0.12)	<0.05
Timor-Leste	−2.07 (−2.14–−2)	<.05	−2.09 (−2.15–−2.01)	<.05	−1.49 (−1.52–−1.47)	<0.05
Turkmenistan	−0.16 (−0.37–0.06)	.14	−0.17 (−0.39–0.06)	.14	−0.07 (−0.1–−0.04)	<0.05
United Arab Emirates	−5.03 (−5.13–−4.92)	<.05	−5.21 (−5.33–−5.09)	<.05	−1.24 (−1.26–−1.22)	<0.05
Uzbekistan	−2.28 (−2.48–−2.06)	<.05	−2.35 (−2.56–−2.13)	<.05	−0.41 (−0.45–−0.38)	<0.05
Viet Nam	−2.74 (−2.82–−2.6)	<.05	−2.89 (−2.98–−2.72)	<.05	−0.48 (−0.51–−0.45)	<0.05
Yemen	−3.71 (−3.86–−3.57)	<.05	−3.77 (−3.93–−3.63)	<.05	−1.08 (−1.1–−1.06)	<0.05

**Figure 2.**
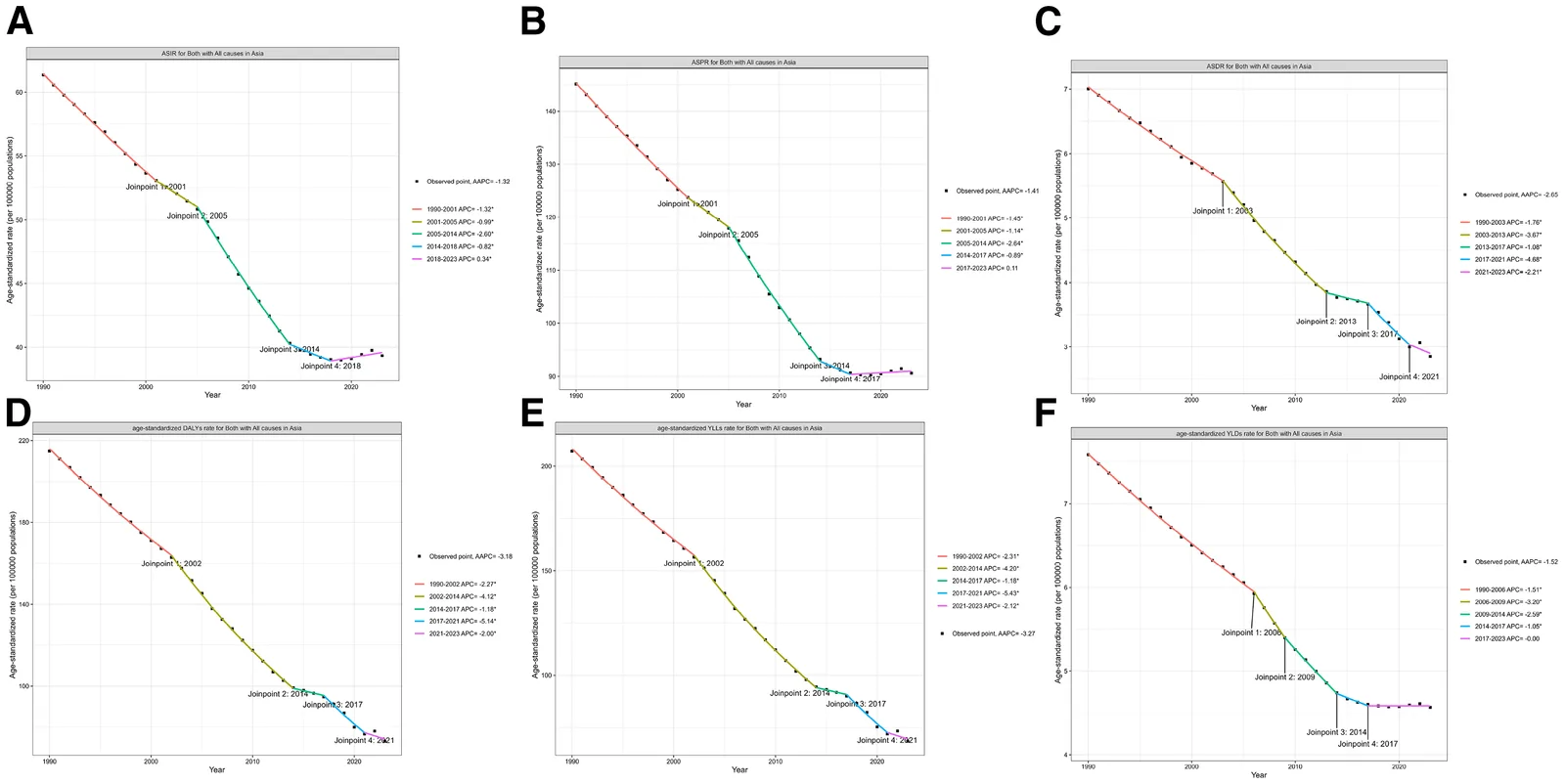
Joinpoint regression analysis of PUD in Asia, 1990 to 2023. (* indicates *P* < .05.) (A) Joinpoint regression analysis of ASIR of PUD in Asia. (B) Joinpoint regression analysis of ASPR of PUD in Asia. (C) Joinpoint regression analysis of ASDR of PUD in Asia. (D) Joinpoint regression analysis of age-standardized DALYs rate of PUD in Asia. (E) Joinpoint regression analysis of age-standardized YLLs rate of PUD in Asia. (F) Joinpoint regression analysis of age-standardized YLDs rate of PUD in Asia. ASDR = age-standardized death rate, ASIR = age-standardized incidence rate, ASPR = age-standardized prevalence rate, PUD = peptic ulcer disease, YLDs = years lived with disability.

### 3.3. Autoregressive integrated moving average

ARIMA projections to 2040 suggest continued decreases in all PUD burden indicators across Asia. The ASIR is projected to decline to 21.85 per 100,000 (95% CI: 0.01–46.97), corresponding to approximately 1.77 million new cases. The wide forecast intervals reflect increasing uncertainty inherent in long-term ecological forecasting; lower bounds approaching zero indicate model-permitted scenarios of substantial reduction but do not imply biological plausibility of zero burden. The ASPR is projected at 48.10 per 100,000 (95% CI: 0.01–107.02), with 4.08 million prevalent cases. The ASDR is projected at 0.65 per 100,000 (95% CI: 0.01–1.29), corresponding to 91,675 deaths. Age-standardized DALYs, YLLs, and YLDs rates are projected at 5.42 (95% CI: 0.01–25.04), 2.23 (95% CI: 0.01–21.68), and 2.79 (95% CI: 0.24–5.35) per 100,000, respectively. Figure 3 displays the projected age-standardized rates of PUD in Asia up to 2040 based on the ARIMA model.

**Figure 3.**
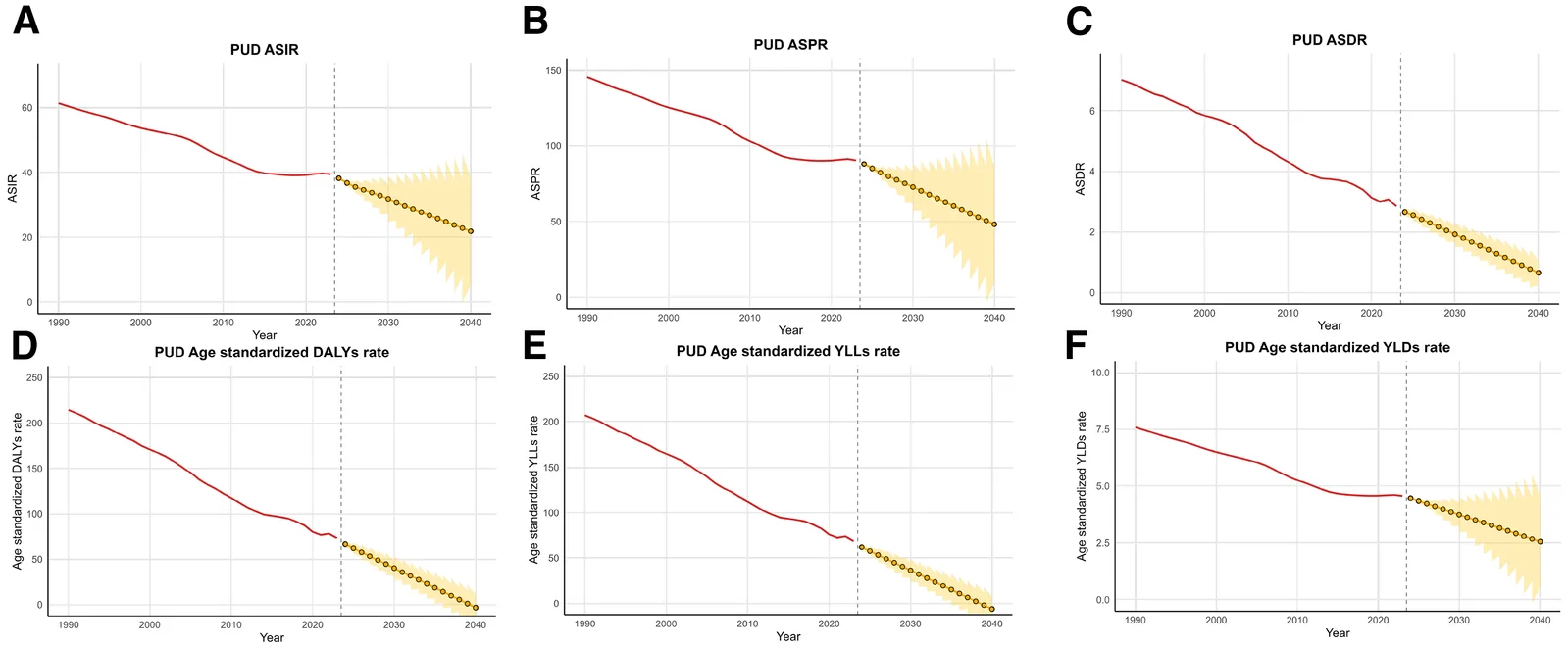
Projected Age-Standardized Disease Burden Rate of PUD in Asia, 2024–2040. (A) Projected ASIR of PUD in Asia. (B) Projected ASPR of PUD in Asia. (C) Projected ASDR of PUD in Asia. (D) Projected Age-Standardized DALYs Rate of PUD in Asia. (E) Projected Age-Standardized YLLs Rate of PUD in Asia. (F) Projected Age-Standardized YLDs Rate of PUD in Asia. ASDR = age-standardized death rate, ASIR = age-standardized incidence rate, ASPR = age-standardized prevalence rate, PUD = peptic ulcer disease, YLDs = years lived with disability.

### 3.4. Interpretation of uncertainty

All reported estimates include 95% UIs reflecting the combined uncertainty from model estimation, input data quality, and parameter variation. At the regional level, the relatively narrow UIs indicate robust estimates driven by comprehensive data coverage across populous countries such as China and India. At the country level, wider UIs in settings with sparse vital registration (e.g., Afghanistan, Yemen, Myanmar) suggest greater uncertainty and warrant cautious interpretation; point estimates for these countries should be viewed as modeled approximations rather than precise measurements. For projections to 2040, the expanding width of forecast intervals over time reflects increasing uncertainty inherent in long-term predictions; lower bounds approaching 0.01 for some metrics indicate that the model permits scenarios of substantial reduction but do not imply biological plausibility of zero burden.

## 4. Discussion

### 4.1. Interpretation of findings

Our study provides the first comprehensive description of the disease burden attributable to PUD across Asia. In 2023, the region recorded almost 1.88 million incident cases and 4.32 million prevalent cases. A prevalence-to-incidence ratio >1 is typical of chronic conditions, which is typical of chronic conditions, confirming that PUD requires long-term, ongoing management.^[18]^ The ASDR from PUD in Asia was 2.85 per 100,000: relatively low. This pattern is consistent with the known effectiveness of modern acid-suppressive therapy and endoscopic interventions in reducing acute mortality from PUD complications such as bleeding and perforation.^[19]^ However, we acknowledge that this interpretation is inferred from the observed mortality decline and existing clinical literature, rather than directly demonstrated by our ecological analysis of GBD modeled data. Our study design does not allow causal attribution of mortality reduction to specific treatments. More than 90% of PUD-related DALYs were attributable to YLLs, whereas YLDs accounted for approximately 6%; Thus, the overwhelming share of PUD’s disease burden stems from premature death rather than from prolonged disability among survivors.^[20]^ Although PUD is often not immediately fatal, complications such as massive hemorrhage or perforation can be rapidly lethal if emergency care is unavailable.^[21]^ Consequently, PUD in Asia is characterized by low overall mortality but high life years lost, indicating that public-health priorities should focus on preventing complications and ensuring prompt emergency treatment. H. pylori eradication and rational use of NSAIDs are the 2 cornerstones of primary and secondary prevention.^[22]^ Expanding population-based H. pylori screening, standardizing treatment protocols, and educating both clinicians and the public on the safe use of NSAIDs represent the most cost-effective strategies for reducing the burden of PUD in Asia.^[23]^

An analysis of the epidemiological trends in Asia’s PUD burden reveals that, although the disease toll has fallen since 30 years ago, a modest upward trajectory has emerged in recent years. Since 2017, incident cases have been rising, driving an increase in prevalent cases after 2018. Encouragingly, the deaths, DALYs and YLLs have continued to decline, underscoring the substantial success of Asian health systems in containing the most severe consequences of PUD. Widespread use of proton-pump inhibitors has been associated with rapid control of bleeding and ulcer healing in clinical trials and cohort studies,^[24]^ while emergency endoscopic hemostasis has become standard practice and has coincided with declining case-fatality rates in population-level data.^[25]^ We emphasize that these observations do not establish causality within our ecological analysis. Yet the expanding incidence and prevalence mean that the pool of patients requiring care is growing, generating sustained demand for outpatient visits, endoscopic procedures and long-term pharmacotherapy. Health systems across Asia must therefore plan and allocate resources accordingly.

Our findings are broadly consistent with previous GBD-based analyses. Xie et al^[14]^ reported global PUD burden trends from 1990 to 2019 and identified South Asia as having the world’s highest prevalence. Bai et al^[15]^ forecasted nonmalignant upper gastrointestinal disease burden globally to 2029, projecting continued declines in PUD-specific metrics. Our Asia-focused analysis extends these studies by providing continent-specific granularity and longer-term projections to 2040. Compared with regional studies, Yang et al^[11]^ reported a PUD prevalence of approximately 10% among Chinese adults aged 18 to 64 years, which aligns with our modeled estimates for China. Choi et al^[12]^ described unchanged overall PUD case numbers in Korea with rising disease in the elderly: a pattern consistent with our age-specific findings showing higher burden in older age groups. Shreya et al^[13]^ documented substantial morbidity and mortality from perforated peptic ulcers in South India, supporting our observation that complications drive the YLL-dominant burden pattern in South Asia.

### 4.2. Policy implications

H. pylori eradication and rational use of NSAIDs are the 2 cornerstones of primary and secondary prevention. Expanding population-based H. pylori screening, standardizing treatment protocols, and educating both clinicians and the public on the safe use of NSAIDs represent the most cost-effective strategies for reducing the burden of PUD in Asia. These recommendations are supported by established clinical evidence and guideline recommendations, though our ecological analysis cannot directly quantify the population-level impact of these interventions.

Based on the ARIMA projections, the burden of PUD is projected to continue declining through 2040, a trend that may coincide with wider H. pylori eradication therapy, advances in endoscopic diagnosis and treatment, and improved public-health awareness. These projections are mathematical extrapolations of historical trends and do not imply causal attribution to specific interventions. However, because of Asia’s huge population and rapid aging, the absolute number of patients needing long-term management would remain substantial. Health systems across Asia must therefore plan resources for chronic drug use, regular follow-ups and repeated endoscopic surveillance.

A notable finding is that YLDs are expected to exceed YLLs in total DALYs by 2040, potentially becoming the dominant component of the disease burden. This reflects that medical progress could substantially reduce premature death from ulcers, while pain, discomfort and reduced quality of life during years lived with the condition would become the core issue. Future priorities should shift from emergency care toward primary prevention: universal H. pylori screening, rational prescribing of NSAIDs: and toward chronic disease management that improves patients’ long-term quality of life and functional status.

## 5. Limitations

### 5.1. This analysis has several important constraints

#### 5.1.1. Ecological study design

Our study analyzed aggregated population-level data, precluding causal inference between specific treatments and PUD outcomes. The observed mortality decline cannot be directly attributed to proton-pump inhibitors, endoscopic therapy, or H. pylori eradication without individual-level data.

#### 5.1.2. Modeled data sources

GBD 2023 estimates are derived from complex modeling (ST-GPR and CODEm) integrating multiple data sources. Their reliability varies across countries depending on underlying data quality and completeness.

#### 5.1.3. Data completeness heterogeneity

Countries with limited vital registration (e.g., Afghanistan, Yemen, Myanmar) have greater uncertainty than high-income settings with robust surveillance (e.g., Japan, South Korea). Urban-biased facility data may inflate estimates, while under-reporting in resource-limited areas may underestimate true burden.

#### 5.1.4. ARIMA forecasting uncertainty

Projections to 2040 assume unchanged trends. Expanding forecast intervals reflect inherent long-term uncertainty; lower bounds approaching zero were constrained by the model variance structure and indicate statistically permissible scenarios of substantial reduction, not biologically plausible zero burden. We did not apply artificial lower-bound constraints; these values emerged naturally from the ARIMA variance estimates. Future interventions or epidemiological shifts could alter trajectories.

#### 5.1.5. Regional aggregation

Aggregating 48 diverse countries masks substantial subregional heterogeneity in burden, healthcare capacity, and data quality.

#### 5.1.6. Temporal lag

GBD 2023 may not fully capture recent disruptions such as the COVID-19 pandemic’s impact on healthcare utilization and PUD diagnosis patterns.

## 6. Conclusion

Although its incidence has declined, PUD continues to impose a substantial disease burden across Asia. The persistent toll: driven by an expanding and aging population: underscores the need for sustained prevention efforts, including population-based H. pylori screening, rational NSAID prescribing, and health system capacity for emergency endoscopic intervention and long-term chronic disease management.

## Author contributions

**Data curation:** Siying Huang, Kaibin Fang, Dunhuang Peng.

**Formal analysis:** Siying Huang.

**Methodology:** Siying Huang, Kaibin Fang, Dunhuang Peng.

**Resources:** Siying Huang.

**Conceptualization:** Kaibin Fang.

**Supervision:** Kaibin Fang.

**Software:** Weitao Hu, Dunhuang Peng.

**Writing – original draft:** Siying Huang, Kaibin Fang, Dunhuang Peng.

**Writing – review & editing:** Siying Huang, Dunhuang Peng.
